# Case of Epilepsy Treated by Tracheotomy

**Published:** 1851-09

**Authors:** W. Cane


*Case of Epilepsy Treated by Tracheotomy.* By W. Cane, Esq.—Dr. Marshall Hall had suggested on several occasions, and especially in conversation with Mr. Cane, that as the attack of epileptic or other convulsion implied closure of the larynx, with expiratory efforts, the attack of convulsive epilepsy would be prevented by tracheotomy. Mr. Cane was summoned, on Feb. 1, 1851, to a boatman, aged 24, who had become subject to violent fits of epilepsy, one of which had just occurred in so extreme a form as to leave him in a state of deep apoplectic coma and asphyxia, inspiration being performed only "by seldom and short catches, whilst the veins of the head and neck were everywhere visible and greatly distended." This state had continued nineteen hours. "Feeling convinced," Mr. Cane observes, "that the patient must shortly expire, and that the root of the evil was in the closure of the larynx, I at once proceeded to open the trachea, a matter of no small difficulty, on account of the twisted state of the neck, the engorged state of the vessels, and the constant action of the muscles. The operation of tracheotomy was performed, and the tracheal tube is kept in the trachea to the present time. The relief to the patient was immediate; the air passed into the lungs, the state of spasm subsided, with the turgid condition of the head and neck, and the patient soon recovered his sensibility. This was not the only gratifying result: although the poor man had experienced his epileptic seizures in increasing violence during seven or eight years, and recently thrice a week, he had not had, up to April 1,—a period of two months,—any return of them. More recent accounts of the patient, who is now in Staffordshire, confirm the former report; the tube is still kept in the trachea and the epileptic seizures have not returned."—*Medical Times.*

